# Prospective Trial of Cerebrospinal Fluid Filtration After Aneurysmal Subarachnoid Hemorrhage via Lumbar Catheter Extension (PILLAR-XT)

**DOI:** 10.1007/s12028-025-02328-8

**Published:** 2025-07-31

**Authors:** Spiros L. Blackburn, Marc A. Babi, Andrew W. Grande, Omar A. Choudhri, Erik F. Hauck, Christopher P. Kellner, Michael C. Giordano, Shivanand P. Lad, Aaron R. McCabe

**Affiliations:** 1 The Vivian L. Smith Department of Neurosurgery, McGovern Medical School, The University of Texas Health Science Center at Houston, Houston, TX USA; 2 Department of Neurosurgery, Neuroscience Institute, Cleveland Clinic, Port St. Lucie, FL USA; 3 Department of Neurosurgery, University of Minnesota Medical School, Minneapolis, MN USA; 4 Department of Neurosurgery, Perelman School of Medicine, University of Pennsylvania, Philadelphia, PA USA; 5 Department of Neurosurgery, Duke University Medical Center, Durham, NC USA; 6 Department of Neurosurgery, Icahn School of Medicine at Mount Sinai, Mount Sinai Health System, New York, NY USA; 7 Minnetronix Medical Inc., Saint Paul, MN USA

**Keywords:** Aneurysm, Subarachnoid hemorrhage, Hydrocephalus, Cerebrospinal fluid, Cerebral hemorrhage

## Abstract

**Background:**

There is a growing consensus that blood in the cerebrospinal fluid (CSF) is deleterious to outcomes in patients with aneurysmal subarachnoid hemorrhage. The extracorporeal filtration of subarachnoid hemorrhage via spinal catheter extension study evaluated the safety, tolerability, and filtration curve of blood and its lysis products from hemorrhagic CSF using the Neurapheresis CSF Management System.

**Methods:**

After aneurysm repair, a dual-lumen intrathecal catheter was inserted into the study participant’s spinal canal. CSF was extracorporeally filtered for up to 72 h, removing blood products from the lumbar cistern, and reintroducing filtered CSF to the thoracic subarachnoid space. Neurological examinations were performed every 2 h, computed tomography scans were captured five times, and CSF samples were evaluated for cell counts every 8 h. Clinical follow-up evaluations were conducted 2 and 30 days after treatment.

**Results:**

Twenty-seven of 29 study participants (93%) had a catheter successfully inserted. The median rate of waste removal was 5.7 mL/hr (interquartile range 3.9–8.8), and the median CSF filtration duration was 37:00 h (interquartile range 24:03–38:52). CSF red blood cell (mean reduction of 86%) and protein cell counts (mean reduction of 82%) decreased much faster in Neurapheresis system–treated participants compared with published data from standard-of-care patients with aneurysmal subarachnoid hemorrhage. From study participant screening through catheter removal, intracranial blood (evaluated via Hijdra Sum Score) decreased by 65%. In four study participants, there were a total of five adverse events, among whom one was determined per protocol to be a serious adverse event. All five events were mild or moderate severity and resolved with no clinical sequelae.

**Conclusions:**

The Extracorporeal Filtration of Subarachnoid Hemorrhage Via Spinal Catheter Extension study demonstrated the potential to significantly accelerate intracranial blood elimination based on imaging (Hijdra Sum Score) and CSF red blood cell and protein reduction measures via a closed-loop filtration system.

## Introduction

Aneurysmal subarachnoid hemorrhage (aSAH) is a life-threatening event resulting in a complex brain injury secondary to acute intracranial pressure changes and delayed neuroinflammation, endothelial cell injury, microthrombosis, and cerebral vasospasm [1–3]. Although treatment and surgical advancements have improved outcomes over time, survivors with aSAH have significant variability in quality of life [1, 4, 5].

Several investigations have identified blood lysis products as a potential physiological cause of poor outcomes in aSAH [6–9]. This concept is supported by data associating increased subarachnoid blood volume and decreased intracranial blood clearance over time (typically measured by the Hijdra Sum Score [HSS]) with poor clinical outcomes [10–14].

Recent American Heart Association guidance for treatment of aSAH updated cerebrospinal fluid (CSF) diversion via external ventricular drain (EVD) or lumbar drain to standard of care [15]. Although data are conflicting, there is evidence that lumbar drainage of intracranial blood improves patient outcomes [16–18]. However, the rate of drainage has been limited in these trials to 5–10 mL/hr to reduce the risk of CSF over drainage and neurological deterioration [16, 18–21]. Filtering blood and blood lysis products out of the CSF and returning filtered CSF to the patient in a closed loop facilitates high volume filtration with a low volume/concentrated waste product. Such a system may provide the benefits of lumbar drainage, but in a more rapid fashion. The Extracorporeal Filtration of Subarachnoid Hemorrhage Via Spinal Catheter Extension (PILLAR-XT) study examined the Neurapheresis CSF Management System’s safety, tolerability, practicality, and capability. The Neurapheresis system is designed to be an automated lumbar drain for external CSF drainage, with the optional capability to filter and return filtered CSF to the body. This study examined the Neurapheresis system’s filtration feature in patients with aSAH following aneurysm treatment.

## Methods

### Study Design and Patient Population

After the successful completion of the first-in-human trial of the Neurapheresis CSF Management System (PILLAR [22]), the system was updated with automation features. Subsequently, the PILLAR-XT study was designed to evaluate the use of the Neurapheresis system in patients with aSAH for a longer filtration period/catheter indwelling duration, to increase the number of study participants from PILLAR, and to provide data to evaluate the system’s safety and CSF filtration ability.

PILLAR-XT was a prospective, single-arm, investigational device exemption study performed at six sites following Federal Drug Administration, central, and local institutional review board approval. Thirty-three study participants with aSAH who met the inclusion/exclusion criteria were consented and enrolled for evaluation of the safety and tolerability of up to 72 h of CSF filtration. Inclusion/exclusion criteria (Table 1) were modeled after typical standard of care for lumbar drainage (moderate/high grade blood, noncomatose, no preexisting or likely future mass effect). The capability of CSF filtration to remove blood and blood byproducts was evaluated by direct lumbar CSF testing and HSS.

**Table 1:** Inclusion/exclusion criteria

*Inclusion Criteria (related to most recent aSAH)*
Male or female patient age: 18–70 years ​
Informed consent by the patient or his/her legally authorized representative​
Modified Fisher Grade 2, 3 or 4 ​
Hunt & Hess I-IV ​
First aneurysmal SAH that has been confirmed by CT scan and secured or planned securement via clipping or coiling per institutional SOC ​
Patient is ≤ 48 h post bleeding event​
World Federation of Neurosurgical Societies (WFNS) Grades I-IV ​
Patient is indicated for a ventriculostomy​^*^
*Exclusion Criteria (related to most recent aSAH)*
Patients with a coagulopathy that cannot be reversed per the professional discretion of the investigator
Pregnancy​
Patients with a SAH due to mycotic aneurysm or arteriovenous malformation​
Patients who present with an acute myocardial infarction or unstable angina​
Patients with uncontrolled diabetes at the time of catheter placement​
Patients who present with a creatinine > 2.0 mg/dl​
Imaging demonstrates supratentorial mass lesions greater than 50 cc^^^
Imaging demonstrates supratentorial mass lesions ≥ 15 cc​^*^
Imaging demonstrates more than 5 mm of mid-line-shift associated with infarction and or edema^^^
Imaging demonstrates ≥ 2 mm of mid-line-shift associated with infarction and or edema​^*^
Imaging demonstrates infratentorial mass lesion ≥ 10 cc​^*^
Imaging demonstrates presence of any subdural hematoma​^*^
Effacement of the basilar cisterns (suprasellar, ambient, chiasmatic and quadrageminal)​
Vasospasm on admission as defined by angiographic evidence​
Patients with a connective tissue disorder that may impact the integrity of the dura​^*^
Thrombocytopenia def. platelet count < 100,000​
Patients on low molecular weight heparin such as Lovenox​
Patients on Clopidogrel bisulfate (Plavix) or other chronic platelet inhibitors​
Patients with a documented history of cirrhosis ​
Non-communicating Obstructive hydrocephalus ​
Patients with a lumbar or thoracic spinal anatomy (e.g. severe spinal stenosis) or history of posterior fusion hardware that would interfere with placement or appropriate in-dwelling of the catheter ​
Existing hardware that prevents accurate CT imaging​
Pre-existing Lumbar Drain ​
Local skin infections or eruptions over the puncture site​
Signs of central nervous system systemic infection, sepsis or pneumonia​
Lumbar puncture within 6 h ​
Concurrent participation in another study which is not observational or retrospective in nature
Without prior approval from the Sponsor​

**^**Criteria only applicable to patients enrolled from 18-Oct-2018 to 11-Mar-2020

*Criteria only applicable to patients enrolled from 11-Mar-2020 until end of trial

The PILLAR-XT study (NCT03607825) was overseen by a data and safety monitoring board (DSMB) consisting of four members who reviewed adverse events: an independent neurointensivist, neurosurgeon, interventional neuroradiologist, and medical ethicist. Adverse events reported in during the trial were adjudicated by an independent medical monitor prior to DSMB review.

### System

**Fig. 1:**
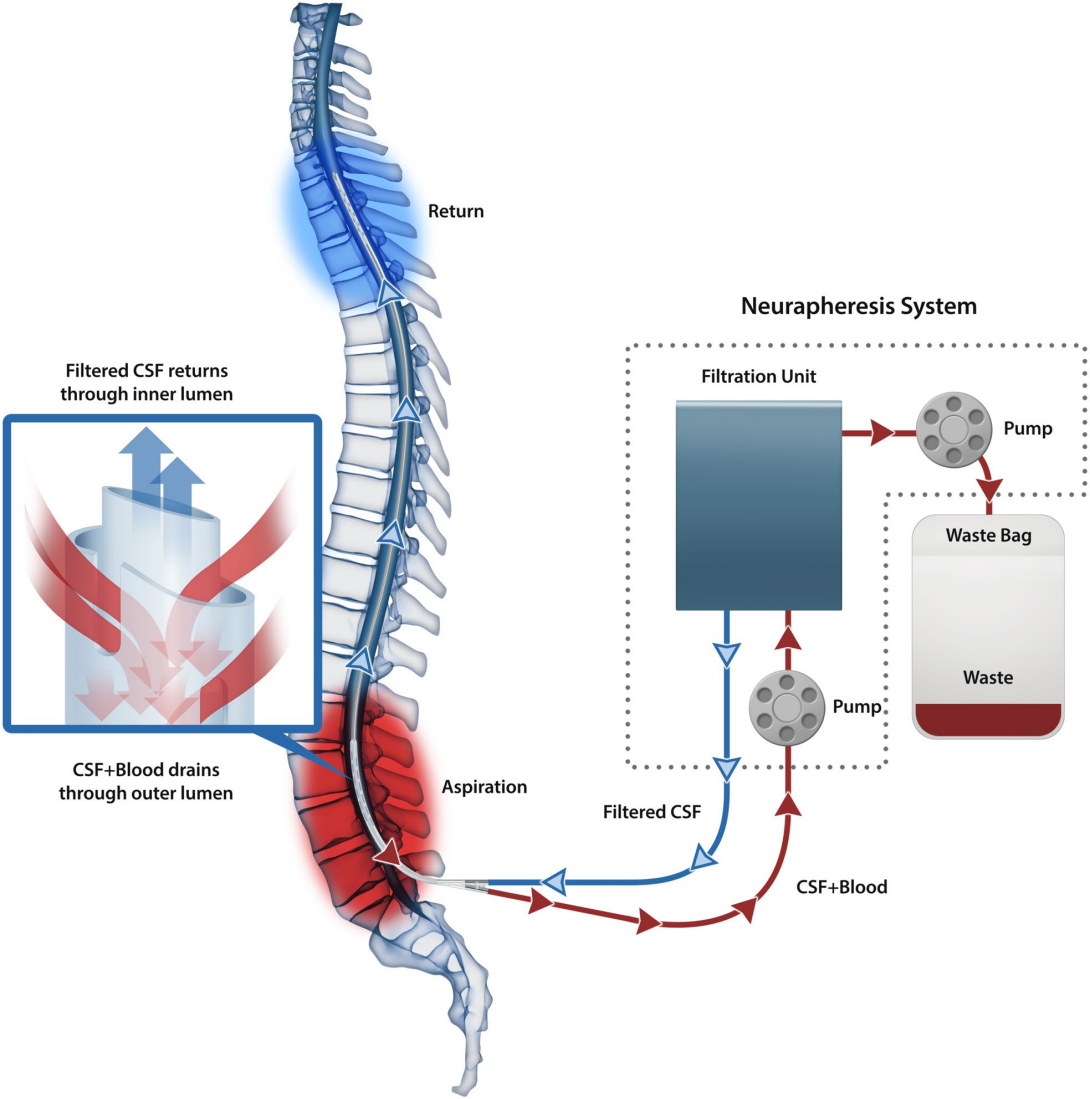
A Graphical representation of the Neurapheresis system. Diagram of the Neurapheresis CSF Management System. CSF with blood and blood byproducts is aspirated from the lumbar region through the outer lumen, filtered, and the filtered CSF is returned to the thoracic region through the inner lumen. The waste products are routed to a waste bag

The Neurapheresis CSF Management System (Fig. 1) uses a dual-lumen catheter placed into L3-L4 or L4-L5 after aneurysm securement, similar to a lumbar drain. The catheter is advanced into the upper thoracic spinal CSF space using over-the-wire technique and fluoroscopic guidance [23]. An extracorporeal filtration unit aspirates CSF from the lumbar subarachnoid space, filters the CSF, and returns the filtered CSF via the second lumen of the catheter to the thoracic region. The distance between the lumbar aspiration ports and the thoracic outlet ports is 30 cm. The waste product (concentrated fluid containing cells, hemoglobin, and lysis products) is routed to a waste bag. The waste rate was configured by the investigator.

During the study, the system was monitored by a field clinical engineer (FCE). Per the investigator’s instruction, the FCE set the waste product rate of the system (e.g., 10 mL/hr of concentrated waste). During the original PILLAR study, the FCE manually adjusted the main filtration rate (which is independent of the waste product rate). In PILLAR-XT, automation replaced manual control of the main filtration rate via monitoring inlet/outlet pressures of the system. While operating within acceptable pressure limits, the automation was designed to slowly, incrementally, increase the main filtration rate to a maximum of 120 mL/hr, while maintaining the waste rate. Per protocol suggestion, the investigators were asked to keep the EVD clamped unless intracranial pressure (ICP) rose above 20 mm Hg or the investigator deemed opening the EVD to be in the best interest of the study participant.

### Neurapheresis Protocol

Per the study design, PILLAR-XT had two phases. The first phase extended the aggregate filtration time (i.e., time the system was actively aspirating, filtering, and returning filtered CSF) from the original PILLAR study maximum of 24 h up to 36 h (± 4 h). Following the successful first phase, a prespecified second phase allowed filtration during the entire catheter indwelling period (up to 72 [± 4] hr). While the catheter was indwelling, neuro checks (ICP, Glasgow Coma Scale [GCS], motor/sensory checks) were performed every 2 h (± 1 h) during the first phase, and every hour (± 1 h) during the second phase. CSF samples including red blood cell (RBC) and protein counts were taken every 8 h.

Predefined potential neurological sequalae were tracked as part of the study including edema, infection, shunt-dependent hydrocephalus (study participant with hydrocephalus who required a shunt), new cerebral infract on imaging (new ischemic event confirmed by computed tomography [CT] or other imaging), new focal neurological deficit (new neurological deficit detected through change in physical attribute), and delayed cerebral ischemia (study participant who had new focal neurological deficit or new cerebral infract on imaging). Neurological and outcome assessments included GCS at 2 days after filtration, modified Rankin Scale, GCS at intensive care unit (ICU) discharge, and Extended Glasgow Outcome Scale, Barthel Index, and study participant location at 30 days.

### CT Scans

Up to five CT scans per study participant were collected via a combination of standard of care and protocol requirements: at screening/enrollment, prior to catheter placement, at 36 h (± 4 h) of catheter indwelling period, immediately after catheter removal, and 2 days (± 1 day) after catheter removal. Study participants had follow-up visits at 2 days (± 1 day) and 30 days (± 3 days) after catheter removal. CT scans were graded by at least two independent neuroradiologists for volume of subarachnoid blood via the Hijdra method, which has been shown to be the most predictive radiological scoring system for complications and outcomes in patients with aSAH [24, 25]. Ten cisternal subscores and four ventricular subscores were each graded 0–3 for amount blood in the region, with 3 being the highest. The following approach was applied to combine the radiologists’ scores for a cistern/ventricle into a single subscore: (1) if at least two radiologists agreed upon a subscore or only one score was available, that subscore was used; and (2) if there was no agreement between radiologists or all scores were missing, the subscore was missing. All subscores were added to calculate the HSS. If a subscore was missing, it was imputed with the mean of all other available subscores, per recommendations [24]. If a study participant was missing all subscores, the sum score was considered missing. Interrater reliability of HSS was evaluated via Spearman’s rank correlation coefficient on samples graded by all three adjudicators.

### Statistical Analysis

Statistical evaluation of CSF blood reduction among PILLAR-XT study participants was performed on HSS and lumbar RBC counts. HSS reduction was compared via a paired *t*-test of HSS for CT scans near-onset (screening/enrollment) and immediately after neurapheresis (catheter removal). Mean reduction of HSS was calculated as the mean of pairwise reductions in HSS between screening/enrollment and catheter removal.

Mean reduction of RBC and protein measurements was calculated as the mean of pairwise reduction between first and last CSF samples. Medians and interquartile ranges (IQRs) of RBC and protein CSF were plotted over time for qualitative analysis of overall trend of the group. For display, study participant CSF values were averaged per study participant within 0.3 days, linearly interpolated, and median/IQR values were derived from available data within a half-day measurement and smoothed. At least 20 study participants had data available for each time point between 1.4 and 3.3 days after onset. One study participant with insufficient RBC and protein CSF samples was excluded from all analysis of RBC and protein CSF. Version 3.9.12 of Python was used for all analyses.

## Results

### Demographics

**Fig. 2:**
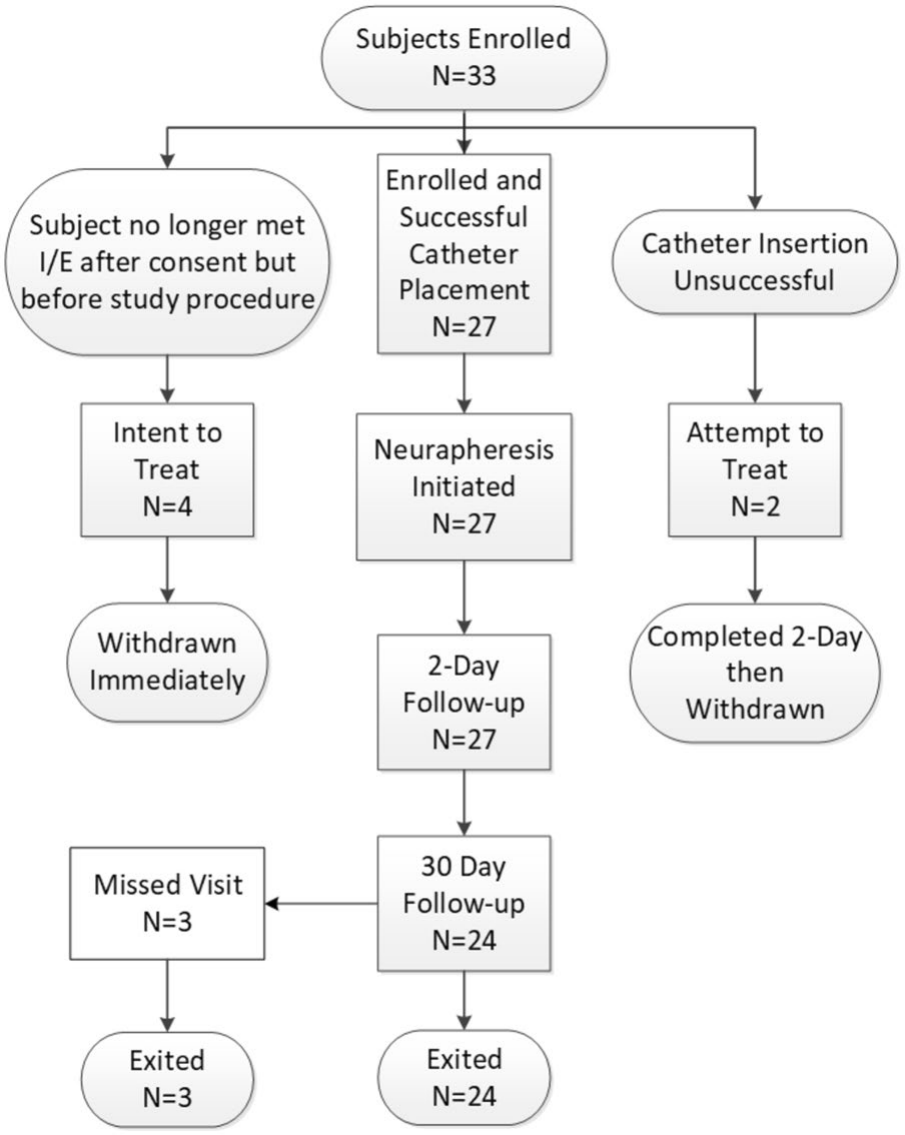
PILLAR-XT study participant flow. CONSORT diagram illustrating the flow of PILLAR-XT participant flow through the study, from enrollment through follow-ups and exit

A total of 33 study participants were enrolled from December 2018 to January 2021. Four study participants consented but catheter placement was not attempted as they were determined to no longer meet inclusion criteria after consent (*n* = 4 intent to treat). These four were withdrawn prior to any study procedure being performed. Of the remaining 29, two study participants had catheter placement attempted but the spinal needle provided with the system could not reach the spinal canal (*n* = 2 attempt to treat). Thus, in total, 27 study participants were enrolled, had successful catheter placement (93% success rate [27/29]), and were treated with CSF filtration via the Neurapheresis CSF Management System (Fig. 2).

All 27 study participants completed the 2-day follow-up visit, and all but three study participants completed the 30-day follow-up visit (*n* = 3 missed visit). An additional study participant’s modified Rankin Scale at ICU discharge was lost.

The mean age of the study participants was 49.9 (± 11.8) years, and the majority were female (22/29, 76%). The median World Federation of Neurological Surgeons scale at admission for these study participants was 2. Eleven of 29 (38%) patients had their aneurysm secured via clipping, whereas the remaining study participants had their aneurysm secured via endovascular coiling. Summary statistics for demographics and admission status can be found in Table 2.

**Table 2:** Demographics and admission status

Parameter​	Pillar-XT (n of N = 29) or mean ± ​standard deviation
Age	49.9 ± 11.8​
Gender (% female)​	76% (22/29)​
Currently smoking	48% (14/29)​
Hypertension	62% (18/29)​
Diabetes​	17% (5/29)​
*Aneurysm securement*	
Clipped	38% (11/29)
Coiled	62% (18/29)​
Both​	0% (0/29)​

Score	Subjects
*Glasgow coma scale (GCS) at admission*	
15	9
14	8
13	0
12	1
11	0
10	5
9	2
8	2
7	2
*Hunt & Hess at admission*	
4	3
3	20
2	5
1	1
*World federation of neurosurgical societies subarachnoid classification at admission*	
4	9
3	5
2	7
1	8
*Modified Fisher scale at admission*	
4	5
3	24

### System Functional Results

Median time from ictus to catheter insertion for CSF management was 34:20 h (IQR 27:08–42:38 h). The median procedure time to successfully place the catheter was 25 min (IQR 21.0–36.5 min) and was achieved on the first attempt in 24/27 successful cases. The length of procedure time per study participant decreased in all sites that gained experience with catheter placement via multiple enrollments.

Median CSF filtration and catheter indwelling time were 37:00 h (IQR 24:03–38:52) and 47:06 h (IQR 43:43–54:04 h), respectively, with a median duty cycle (filtration time divided by catheter indwelling time) of 76% (IQR 57–85%). The median waste product rate, the concentrated equivalent of “drain rate” in a traditional lumbar drain, was 5.7 mL/hr (IQR 3.9–8.8 mL/hr) with a median total waste product removed of 357.5 mL (IQR 185.5–428.3 mL). The median filtration rate was 40.8 mL/hr (IQR 34.2–48.6). Summary statistics for catheter placement and treatment can be found in Table 3.

**Table 3:** Catheter placement and CSF management statistics

Catheter placement​ and CSF management statistics	Median [Q1-Q3] or % (n/N)​
Hours from onset to catheter securement​	34:20 h ​[27:08–42:38]​
Catheter indwelling time	47:06 h​ [43:43–54:04]​
Catheter placement success​	93% (27/29)​
Length of procedure time (all attempts) (minutes)​	25 min [21.0–36.5]​
Length of procedure time (first attempts) (minutes)​	24 min [20–31]​
Total cerebrospinal fluid (csf) filtration time (hrs)​	37:00 h​ [24:03–38:52]​
Filtration rate (mL/hr)	0.68 mL/min [0.57–0.81]
Waste volume removed (mL)​	357.50 ml​ [185.50–428.30]​
Waste rate (mL/hr)​	5.7 ml/hr​ [3.9–8.8]​

### Blood and blood lysis product dynamics

**Fig. 3:**
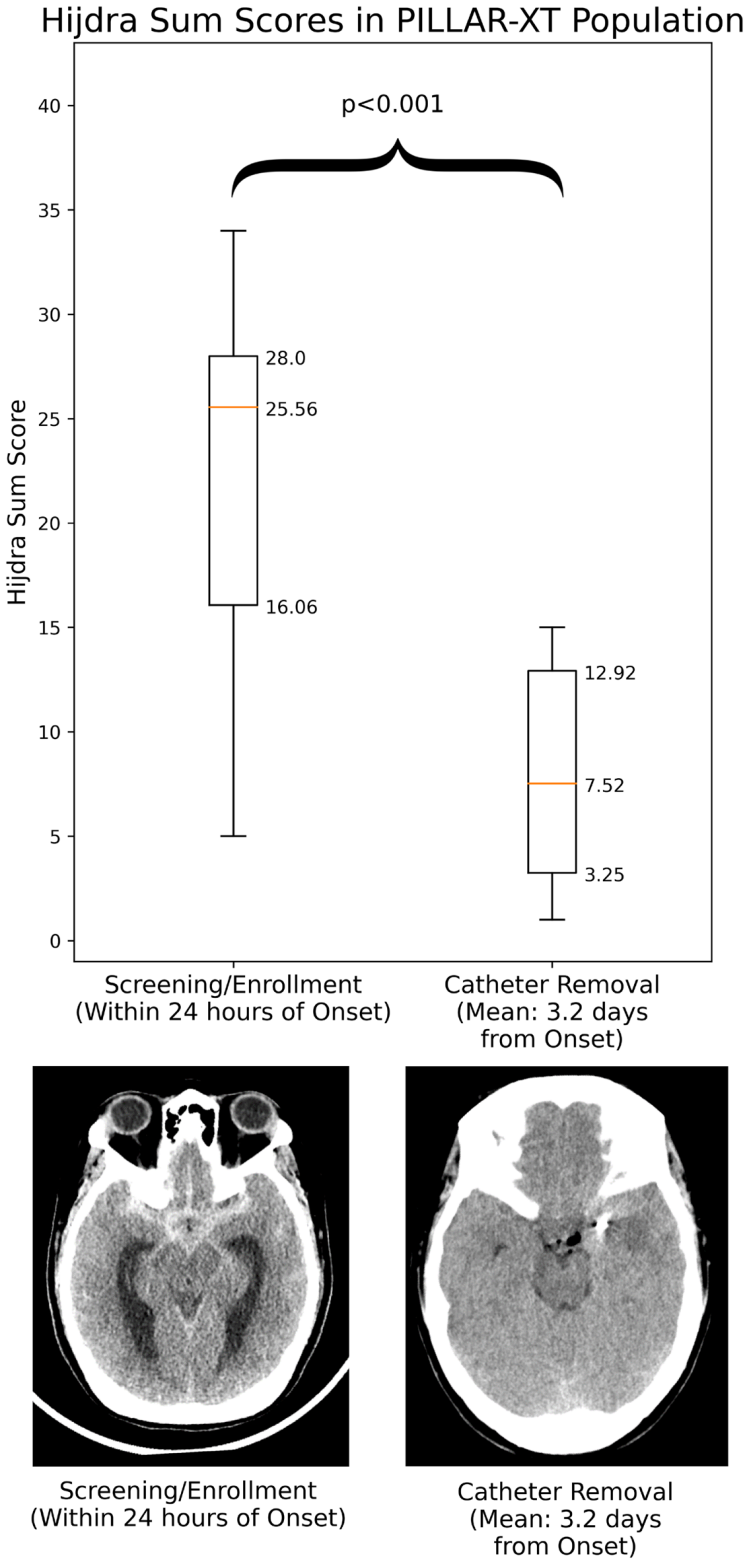
Hijdra Sum Score before and after CSF filtration. Distribution of Hijdra Sum Score for treated PILLAR-XT study participants at two timepoints: Near-Onset (Screening/Enrollment) and Immediately Post-Neurapheresis (Catheter Removal). Hijdra Sum Score decreased between these time points across all study participants. One study participant’s CT scan at both time points is shown in the bottom panel of the figure for reference. This study participant is a fair representation of the median patient: their HSS reduced from 24 at screening/enrollment to 8 at catheter removal. One study participant with missing data is not shown. One study participant with data located outside the whiskers (shown as Q1 or Q3 ± 1.5*IQR) is not shown. A paired t-test comparing screening to catheter removal yielded *p* < 0.001

Median HSS among study participants receiving CSF filtration decreased from 25.6 at patient enrollment to 7.5 immediately after catheter removal. The mean reduction of HSS among study participants between enrollment to catheter removal was 65%. The median and distribution of HSSs in study participants receiving Neurapheresis across these two time points is illustrated in Fig. 3. Spearman’s rank correlation coefficient for HSS across adjudicators was 0.95 (adjudicator A to adjudicator B), 0.94 (A–C), and 0.92 (B–C).

**Fig. 4:**
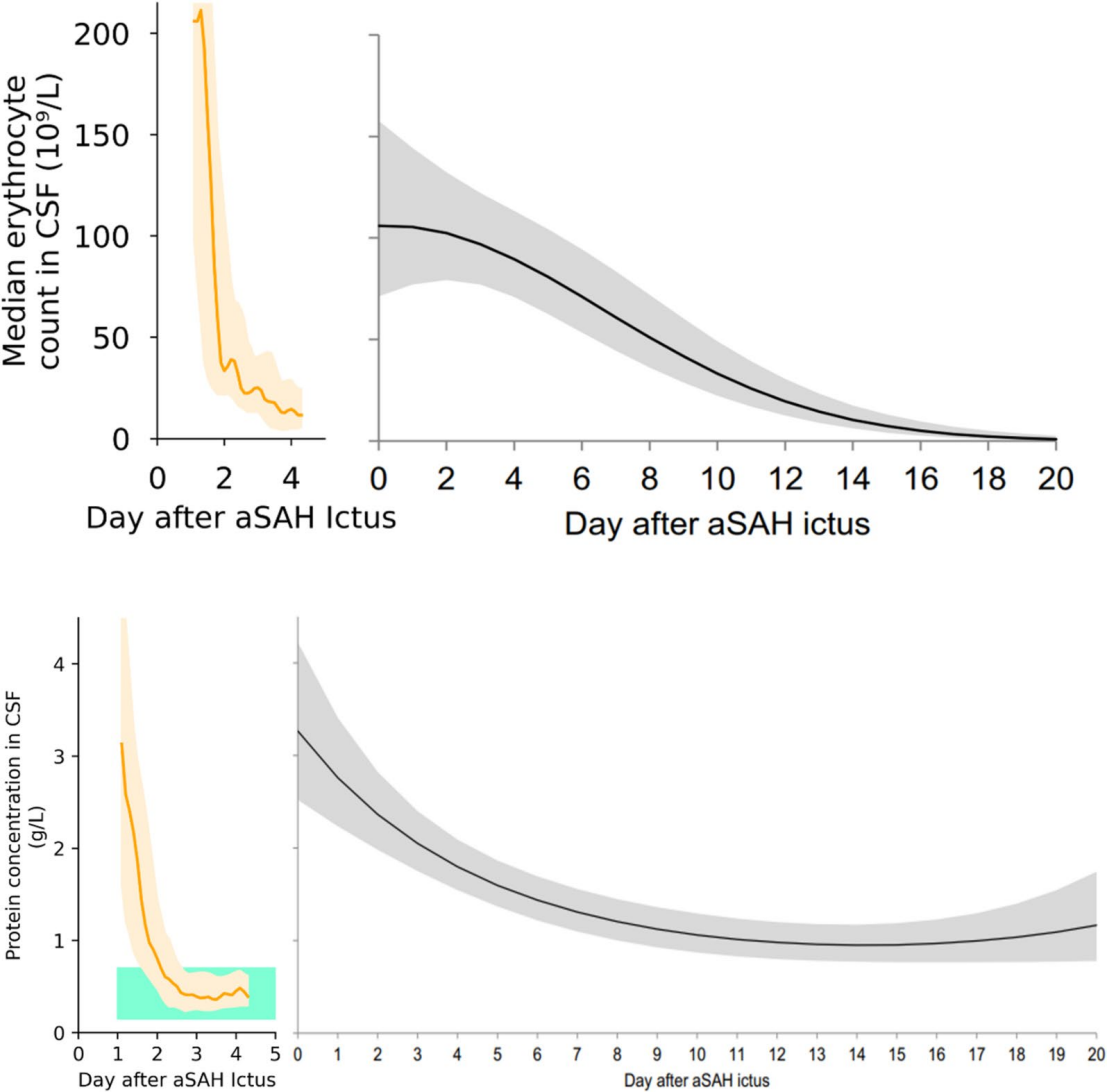
RBC Clearance in PILLAR-XT study participant and protein clearance in PILLAR-XT study participants. **A**, **B**: Course of RBC (**A**) and Protein Clearance in PILLAR-XT study participants (**B**). Protein and RBC measurements decreased rapidly during the period CSF filtration was active (1.5–3.4 days post-onset on average across study participants). Normal CSF protein range shown is 0.15–0.7 g/L [26]. Shaded regions are IQR for PILLAR-XT study participants. Figure 4C (top right): RBC Clearance as Reported by Koopman, et al. and Fig. 4D (bottom right): Protein Clearance as Reported by Koopman et al. **C**, **D**: Course of RBC (**C**) and Protein Clearance (**D**) in treated per standard of care over their clinical course [32]. Normal CSF protein range shown is 0.15–0.7 g/L [26]. Shaded regions are 95% confidence interval for Koopman et al. [32]

CSF RBC and protein counts in PILLAR-XT study participants were significantly reduced over time post-onset (paired *t*-test, *p* = 0.003; Fig. 4a and b). CSF RBC counts reduced from a mean of 540 × 10^9^ cells/L in the first measurement to a mean of 30 × 10^9^ cells/L in the last measurement. CSF total protein concentration reduced from a mean of 3.99 g/L in first CSF measurements to a mean of 0.56 g/L in last measurements. Mean CSF RBC and protein reductions from first to last measurement across the study participants were 86% and 82%, respectively. Twenty of 26 study participants returned to normal (0.15–0.7 g/L) protein range within 5.5 days of SAH onset [26]. One study participant outside this range was measured slightly lower (0.13 g/L) than the normal protein range.

### Study Participant Clinical Trajectory/Outcomes

Twenty-five of 27 study participants (92%) maintained or improved their GCS score from admission to ICU discharge, and 16 of 25 study participants (64%) were located at home at 30 days. Twenty-four of 27 study participants did not have delayed cerebral ischemia. Predefined potential neurological sequelae, location at 30 days, and neurological assessments at ICU discharge and 30 days are summarized in Table 4.

**Table 4:** Outcome statistics

Outcome statistics	Median [Q1-Q3] or % (n/N)​
New focal neurological disorder​	11%​ (3/27)
New cerebral infract on imaging​	7%​ (2/27)
Delayed cerebral ischemia	11%​ (3/27)
Edema​	11% (3/27)​
Seizure	4%​ (1/27)
Central nervous system infection​*	0%​ (0/27)
Shunt dependent hydrocephalus​	19%​ (5/27)

Score	Number of subjects
*Glasgow coma scale (GCS) at 2-days post neurapheresis*	
15	16
14	3
13	2
11	2
10	2
8	1
7	1
Modified Rankin score (mRS) at ICU discharge**	
5	4
4	6
3	1
2	3
1	11
0	1
GCS at ICU discharge	
15	18
14	2
13	3
11	1
10	2
8	1
*Glasgow outcome scale extended at 30-days*^*^*^	
8	1
7	9
6	3
5	2
4	3
3	6
Barthel index at 30-days^^^	95 [60–100]
mRS at 30-days^^^	
5	2
4	5
3	4
2	3
1	9
0	1
Location home @ 30-days^^^^	64% (16/25)
Location intermediate (rehab, stepdown, still-hospitalized, SNF) @ 30-days^^^^	28% (7/25)
Location ICU @ 30-days^^^^	8% (2/25)

*Within 5 days post Neurapheresis catheter removal, per protocol pre-defined analysis

**Missing one subject’s data due to protocol deviation

^^^Missing three subject’s data due to lost follow-up

^^^^Although only 24 subjects had a complete 30-Day follow-up, one additional subject’s location was known at this time point and thus is included for completeness

In four study participants, there were a total of five adverse events, one of which was determined per the protocol to be a serious adverse event (SAE). All five were graded by the IMM and confirmed by the DSMB to be of mild or moderate severity and resolved without clinical sequelae. Two adverse events were localized pain in the legs or back, and two were headaches, both of which are typical in the patient population with spinal catheter placement and in aSAH in general. One study participant’s treatment was suspended due to early signs of transtentorial herniation, and reversed using standard clinical protocol, with complete resolution of symptoms and no clinical sequelae.

## Discussion

Aneurysmal subarachnoid hemorrhage is a devastating neurological condition that imposes a severe burden on the individual and the public due to its mortality rate, disability rate, incidence among younger individuals [27], and large financial burden on health care resource utilization [28]. Physiological mechanisms of delayed deterioration owing to aSAH, and interventions to enhance outcomes for these individuals are an active area of study [29–31]. In this study, we investigated a novel device designed to filter blood from the CSF and operate within the ICU environment similarly to a lumbar drain.

This study demonstrated the potential for the Neurapheresis CSF Management System to safely manage, filter, and return CSF in 27 study participants. During and after filtration, blood and protein concentrations in the CSF were dramatically lowered in the lumbar space across the cohort. Furthermore, cranial blood, as measured by the HSS, was also substantially lowered in this group. In the lumbar region, the mean decreases from first to last lumbar CSF sample were 86% for RBCs and 82% for total proteins in the CSF, which contrast with a recently published study by Koopman et al. [32] (Fig. 4c and d) examining patients with aSAH with either an EVD (61%) or lumbar drain (39%) as a means to manage CSF. It is difficult to compare the lumbar CSF counts from PILLAR-XT with Koopman et al. [32] with exactness, as ventricular CSF and lumbar CSF counts can differ [33]. Nevertheless, study participants in the PILLAR-XT study having a higher initial median RBC count than those in Koopman et al. [32], PILLAR-XT study participants reached the same median RBC CSF levels in 1.8 days after ictus compared with 7 days in those with standard of care (Fig. 4a and c) [32]. CSF protein concentrations between the groups began at roughly equivalent points (median 3.3 g/L at day 0 after ictus in Koopman et al. [32] vs. 3.13 g/L at 1.1 days after ictus in study participants receiving CSF filtration) but also quickly diverged (Fig. 4b and d). Median protein concentration in study participants treated with the Neurapheresis system reached normal levels (< 0.7 g/L) in 2.1 days after ictus, whereas standard-of-care patients median protein CSF concentrations never returned to normal levels in 20 days after ictus [26].

The original PILLAR trial (*n* = 13) relied on manual control of the filtration rate by an FCE for a limited amount of time (up to 24 h). PILLAR-XT (*n* = 27) replaced manual control of the filtration with a pressure monitoring based algorithm and extended the amount of filtration time to up to 72 h. Longer filtration times (up to 72 h) and higher utilization of the system (76% median duty cycle) in PILLAR-XT when compared with PILLAR (up to 24 h, 53% median duty cycle) led to an increase in total CSF processed for PILLAR-XT study participants (PILLAR-XT mean = 1530 mL, PILLAR mean = 632 mL). As such, HSS reduction in PILLAR-XT (65%) outpaced the HSS reduction in PILLAR (47%). Similarly, mean CSF RBC counts after catheter removal were much lower in PILLAR-XT (30 × 10^9^ cells/L) compared with PILLAR (177 × 10^9^ cells/L). This finding supports the hypothesis that filtration time directly impacts subarachnoid blood clearance. A comparison of CT imaging of SAH and outcomes of study participants in the PILLAR-XT trial with a representative standard-of-care population is currently underway.

The mild or moderate adverse events in the study (headache and pain) were not unexpected after aSAH or after lumbar catheter placement. The one SAE was determined by the DSMB to be associated with a postcraniotomy aneurysm clipping study participant at higher risk for herniation, who might not otherwise have been considered for traditional lumbar drainage. Following this SAE, the inclusion/exclusion criteria were adjusted and there were no additional occurrences in the remainder of the study.

The interpretation of these results may be limited by several factors. The study does not directly compare to EVD or lumbar drain RBC/protein levels in the CSF. However, there are strong historical records (Koopman et al. [32]), which provide evidence that CSF filtration via the Neurapheresis system reduces RBCs/proteins faster than other approaches, although historical records include a pooled mixture of patients with an EVD (61%) and lumbar drain (39%). Another potential limitation in this analysis is the utilization of EVDs for drainage during the period of Neurapheresis filtration. Although the protocol suggested keeping the EVD clamped unless ICP rose above 20 mm Hg, it also allowed for the investigator to open the EVD if deemed in the best interest of the study participant. EVDs were opened in 11 of the 27 study participants, although in a majority of the cases, this was temporary. EVD output was not tracked during these periods. Although this study did not evaluate the impact of CSF filtration on clinical outcome, recent evidence suggests CSF drainage, HSS, and natural clearance of blood in the aSAH population all are associated with improved patient outcomes [13, 16, 34]. Given the greater blood reduction capability demonstrated by the Neurapheresis system in this study, the potential exists for CSF filtration via the Neurapheresis device to filter more blood immediately after aneurysm securement than via lumbar or ventricular drainage alone. A future comparison with EVD and lumbar drain as published in the positive EARLYDRAIN trial would be valuable to further explore these results [16].

## Conclusions

The PILLAR-XT study demonstrates the safety and capability of a novel filtration device to remove blood, blood breakdown products, and protein from the CSF. This device has the potential to significantly accelerate the elimination of blood and blood breakdown products from CSF in patients with aSAH.

## Declarations

### Clinical Trial Registration

ClinicalTrials.gov: NCT03607825.
