# Correction: Prospective Trial of Cerebrospinal Fluid Filtration After Aneurysmal Subarachnoid Hemorrhage via Lumbar Catheter Extension (PILLAR-XT)

**DOI:** 10.1007/s12028-025-02380-4

**Published:** 2025-09-08

**Authors:** Spiros L. Blackburn, Marc A. Babi, Andrew W. Grande, Omar A. Choudhri, Erik F. Hauck, Christopher P. Kellner, Michael C. Giordano, Shivanand P. Lad, Aaron R. McCabe

**Affiliations:** 1 Department of Neurosurgery, McGovern Medical School, The University of Texas Health Science Center at Houston, Houston, TX USA; 2 Department of Neurosurgery, Neuroscience Institute, Cleveland Clinic, Port St. Lucie, FL USA; 3 Department of Neurosurgery, University of Minnesota Medical School, Minneapolis, MN USA; 4 Department of Neurosurgery, Perelman School of Medicine, University of Pennsylvania, Philadelphia, PA USA; 5 Department of Neurosurgery, Duke University Medical Center, Durham, NC USA; 6 Department of Neurosurgery, Icahn School of Medicine at Mount Sinai, Mount Sinai Health System, New York, NY USA; 7 Minnetronix Medical Inc., Saint Paul, MN USA

**Correction to: Neurocritical Care** 10.1007/s12028-025-02328-8

The article Prospective Trial of Cerebrospinal Fluid Filtration After Aneurysmal Subarachnoid Hemorrhage via Lumbar Catheter Extension (PILLAR‑XT), written bySpiros L. Blackburn, Marc A. Babi, Andrew W. Grande, Omar A. Choudhri, Erik F. Hauck, Christopher P. Kellner, Michael C. Giordano, Shivanand P. Lad and Aaron R. McCabe, was originally published under exclusive license to 2025 Springer Science+Business Media, LLC, part of Springer Nature and Neurocritical Care Society. As a result of the subsequent decision to publish the article under the open access model, the article’s copyright notice was changed on 29 August 2025 to © The Authors 2025 and the article is now distributed under a Creative Commons Attribution.

This article is licensed under a Creative Commons Attribution 4.0 International License, which permits use, sharing, adaptation, distribution and reproduction in any medium or format, as long as you give appropriate credit to the original author(s) and the source, provide a link to the Creative Commons licence, and indicate if changes were made.

The images or other third party material in this article are included in the article’s Creative Commons licence, unless indicated otherwise in a credit line to the material. If material is not included in the article’s Creative Commons licence and your intended use is not permitted by statutory regulation or exceeds the permitted use, you will need to obtain permission directly from the copyright holder.

To view a copy of this licence, visit http://creativecommons.org/licenses/by/4.0

